# The Cavendish Lecture

**Published:** 1912-07-06

**Authors:** Karl Pearson


### The Cavendish Lecture.

*To the Editor of* The Hospital.

Sir,—In a note last week on the Cavendish Lecture you use the following words: "We think Professor Pearson is on much less safe ground when he deprecates efforts to lessen infantile mortality." I should be glad to know on what grounds you base the statement that "I deprecate efforts to lessen infantile mortality." I do not think there is a single word in the Cavendish Lecture which justifies this statement, and there is much directly opposed to it. I directly asserted the right of everyone born to live, and praised highly the medical profession for assisting in the process. What I did contest was the right of everyone born to reproduce their kind, which is a very different matter.

The Cavendish Lecture will be issued in a few days, and, I think, then, Sir, it will be clear how little ground there is for the above statement. The dissemination of that view is due to a perfectly well-known origin, a writer who believes that he can discredit the work done in this Laboratory by asserting that it holds a "better dead" principle, whereas we have ever upheld a quite different view of life, with which most medical men of experience concur, the "better-not-born" principle. The fact that the death-rate is selective is a scientific fact, and nothing is gained by screening it. It is indeed the strongest argument that can be raised in favour of a eugenic policy. If we keep, as we must keep, the weaklings alive, we must at the same time see that the maximum fertility is not that of the degenerate stocks. This was the essential doctrine of my recent Cavendish Lecture.—I am, Sir, etc.,

June 28. Karl Pearson.

[The grounds upon which our statement was based were the general impressions formed by listening to the lecture. If those impressions are erroneous, we are glad to hear it; and the fault must lie either in our own perceptions or in the Professor's exposition.—Ed. The Hospital.]

